# Positive intratumoral chemokine (C-C motif) receptor 8 expression predicts high recurrence risk of post-operation clear-cell renal cell carcinoma patients

**DOI:** 10.18632/oncotarget.6761

**Published:** 2015-12-24

**Authors:** Qiang Fu, Yuan Chang, Lin Zhou, Huimin An, Yu Zhu, Le Xu, Weijuan Zhang, Jiejie Xu

**Affiliations:** ^1^ Department of Biochemistry and Molecular Biology, School of Basic Medical Sciences, Fudan University, Shanghai, China; ^2^ Department of Urology, Zhongshan Hospital, Fudan University, Shanghai, China; ^3^ Department of Urology, Shanghai Cancer Center, Fudan University, Shanghai, China; ^4^ Department of Urology, Ruijin Hospital, School of Medicine, Shanghai Jiaotong University, Shanghai, China; ^5^ Department of Immunology, School of Basic Medical Sciences, Fudan University, Shanghai, China

**Keywords:** chemokine (C-C motif) receptor 8, clear-cell renal cell carcinoma, prognostic factor, recurrence-free survival

## Abstract

Chemokine (C-C motif) receptor 8 (CCR8) could drive cancer progress through recruiting certain immune cells. Recent evidences revealed the chemotaxis of CCR8^+^ human malignant tumor cells towards lymph node, and a significantly increased CCR8 expression in renal carcinomas patients. To assess the clinical association between CCR8 expression and the risk of post-surgery recurrence in patients with clear-cell renal cell carcinoma (ccRCC), we detected intratumoral CCR8 expression in 472 post-nephrectomy ccRCC patients retrospectively enrolled. Positive CCR8 staining tumor cell occurred in 26.1% (123 of 472) non-metastatic ccRCC cases, and the positive expression was associated with increased risks of recurrence (Log-Rank *P* < 0.001). In multivariate analyses, CCR8 expression was identified as an independent prognostic factor (*P* = 0.008) and entered into a newly-built nomogram together with T stage, Fuhrman grade, tumor size, necrosis and lymphovascular invasion. Calibration curves showed optimal agreement between predictions and observations, while its C-index was higher than that of Leibovich score for predicting recurrence-free survival (RFS) of localised RCC patients (0.854 *vs* 0.836, respectively; *P* = 0.044). The practical prognostic nomogram model may help clinicians in decision making and design of clinical studies.

## INTRODUCTION

Chemokine (C-C motif) receptor 8 (CCR8), one member of the C-C motif chemokine receptor superfamily, was initially identified in activated T helper type 2 (Th2) cells as a receptor of CCL1/I-309 [1, 2]. The CCL1-CCR8 axis is involved in the pathological processes of various inflammatory diseases. This chaining is believed to mediate Th2 cell and Treg cell recruitment in allergic inflammation such as asthma and atopic dermatitis. CCR8^+^ macrophages also play significant roles in postoperative peritoneal adhesion development [3]. The involvement of CCR8 in these diseases suggests that it is a unique modular in inflammatory/allergic responses by inducing tissue damage and remodeling. Recently some studies focusing on the behavior of CCR8 in carcinogenesis were conducted and have implicated an anti-apoptosis effect of CCR8 in lymphoma and T cell leukemia through an autocrine manner [4]. Unexpectedly, Suvendu et al reported that CCL1 produced by lymphatic endothelial cells could facilitates CCR8^+^ tumor cell entry into the open subcapular sinus as well as subsequent migration into the lymph node cortex [5]. These data identify a novel mechanism of regulation of tumor cells migration by virtue of CCR8. However, whether CCR8 plays a role in clinical recurrence of patients with solid tumors is not yet determined.

Renal cell carcinoma (RCC) represents 3∼4% of adult solid tumors. It is frequently diagnosed with synchronous or metachronous metastases and has an estimated age-standardized mortality in Europe of 2.6% [6]. The major histologic subtypes of RCC is clear-cell RCC (ccRCC, 75-85%), of which more than one tenth patients would occur fatal recurrence within 5 years after traditional partial or radical nephrectomy. Although pathologic factors such as coagulative necrosis, metastatic status, sarcomatoid features, and lymphovascular invasion (LVI) have been extensively addressed, their impacts on ccRCC prognosis are however inconclusive [7]. Following surgery for localised RCC some recent studies rely on models combining standard models with molecular biomarkers to make progress in better stratifying recurrence risk to aid patient counselling, personalize follow-up, and target adjuvant treatment trails [8, 9]. Herein sustained studies to evaluate the prognostic value of genetic and proteome signature in RCC patients are unremittingly on going.

Previous works by Evgeniy et al have indicated that CCR8^+^ myeloid cell subset is expanded in RCC patients and that their accumulation composes cancer-related inflammation and could contribute to tumor cells immune evasion during metastatic cascade by means of Treg cells aggregation. Furthermore, blockage of CCL1-CCR8 signals may provide an attractive strategy for potential therapeutic intervention [10]. In view of the likelihood of CCR8 expression on tumor cells as it was reported to trigger tumor cells migration or invasion through lymphatic vasculature, and the open data from The Cancer Genome Atlas that CCR8 mRNA are unregulated in 16% ccRCC cases (61 of 392), we hypothesized that intratumoral overexpression of CCR8 may serve as a potential prognosticator of ccRCC recurrence.

In this study, we analyzed the expression of intratumoral CCR8 and the impact of CCR8 expression on recurrence-free survival (RFS) in a large cohort of ccRCC patients without synchronous metastatic diseases.

## RESULTS

### Clinical and pathologic characteristic

**Figure 1:**
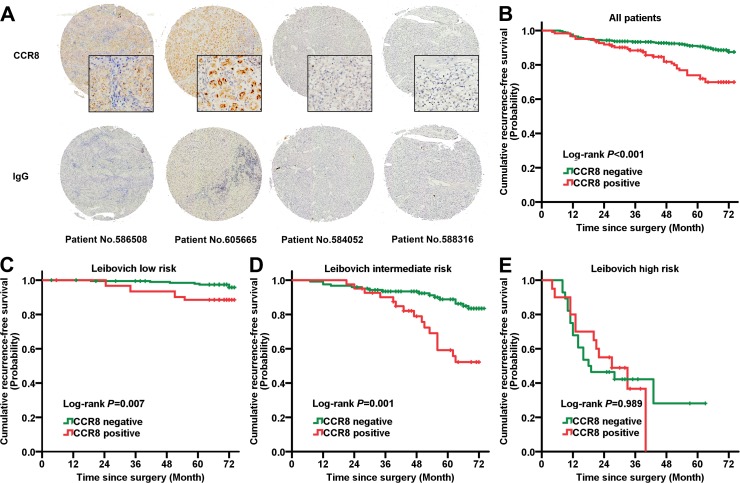
Prognostic power of CCR8 in diverse Leibovich risk groups **A**. Typical immunohistochemistry staining images of CCR8 and isotype IgG in ccRCC tumor tissues. **B**. Kaplan-Meier analysis of RFS in entire ccRCC patients according to intratumoral CCR8 expression. **C**.-**E**. Kaplan-Meier analysis of RFS according to intratumoral CCR8 expression in **C**. Leibovich low risk, **D**. Leibovich intermediate risk, **E**. Leibovich high risk patients. Abbreviation: RFS, recurrence-free survival; CCR8, CC chemokine receptor 8.

After applying initial exclusion criteria, a total of 472 patients with pT1-3N0M0 ccRCC underwent nephrectomy from 2008 to 2009. Mean age in the entire group was 55.1 yr (range 21-86). Of these patients, 123 patients (26.1%) had positive CCR8 tissue staining (Figure 1A). Table 1 compares their clinical and pathologic features. Overall, no significant difference of age and gender between CCR8^+^ and CCR8^−^ groups, while higher Fuhrman grade, presence of LVI and necrosis were significantly associated with positive CCR8 expression (*P* = 0.017, 0.005, 0.002, respectively).

**Table 1:** Correlations between CCR8 expression and clinical characteristics in non-metastatic ccRCC patients

			CCR8				
	All patients (*n* = 472)		Negative (*n* = 349)		Positive (*n* = 123)		
Variables	Cases (%)	6-yr RFS	Cases	6-yr RFS	Cases	6-yr RFS	*P*‡
Age at surgery, yr							
Median (IQR)	55 (46 to 63)		55 (46 to 63)		56 (47 to 61)		0.895†
≤55	239 (50.5%)	83.3±3.0	179	85.9±3.3	60	75.0±6.1	S
>55	233 (49.5%)	82.5±2.6	170	89.1±2.5	63	65.3±6.3	S
Gender							0.134*
Female	137 (29.0%)	86.1±3.2	108	91.8±2.8	29	64.4±9.7	S
Male	335 (71.0%)	81.6±2.5	241	85.5±2.8	94	71.6±5.0	S
ECOG-PS							0.261*
0	393 (83.3%)	87.6±1.9	295	90.9±2.0	98	78.1±4.3	S
≥1	79 (16.7%)	51.9±7.6	54	64.9±8.2	25	19.9±12.1	S
Surgery							0.676*
Partial nephrectomy	227 (48.1%)	88.6±2.2	170	90.6±2.3	57	82.1±5.5	S
Radical nephrectomy	245 (51.9%)	77.9±3.0	179	84.8±3.2	66	59.5±6.5	N
Tumor size, cm							
Median (IQR)	4.0 (2.6 to 5.0)		4.0 (2.9 to 5.0)		3.5 (2.5 to 5.5)		0.805†
≤4.0	296 (62.7%)	89.2±2.1	214	93.6±2.1	82	78.2±4.7	S
>4.0	176 (37.3%)	71.3±3.9	135	77.1±4.0	41	48.9±9.7	S
Pathological T stage							0.518*
pT1	330 (69.9%)	88.3±2.0	244	91.9±2.1	86	78.1±4.6	S
pT2	33 ( 7.0%)	78.6±7.2	27	80.0±8.0	6	77.8±17.9	N
pT3	109 (23.1%)	71.1±4.7	78	79.0±5.0	31	49.9±9.9	S
Fuhrman grade							0.017*
1	90 (19.1%)	94.3±2.5	66	96.9±2.1	24	87.3±6.9	S
2	217 (46.0%)	92.2±1.9	172	95.6±1.6	45	79.4±6.1	S
3	107 (22.6%)	70.6±6.9	77	76.4±7.7	30	56.2±11.2	S
4	58 (12.3%)	41.3±7.7	34	40.2±10.0	24	43.6±12.0	N
LVI							0.005*
Absent	353 (74.8%)	89.0±2.0	273	91.5±2.1	80	80.4±4.7	S
Present	119 (25.2%)	63.7±4.9	76	72.8±5.5	43	49.6±8.3	N
Sarcomatoid features							0.804*
None	459 (97.2%)	84.3±1.9	339	89.2±2.0	120	70.4±4.4	S
Present	13 ( 2.8%)	23.1±17.7	10	40.0±15.5	3	66.7±27.2	N
Coagulative necrosis							0.002*
None	377 (79.9%)	88.5±1.9	291	90.8±2.1	86	81.1±4.4	S
Present	95 (20.1%)	58.9±5.7	58	70.8±6.3	37	38.1±9.6	S
Follow-up duration, mo							
Median (IQR)	73.0 (72.0 to 74.0)		73.0 (72.0 to 74.0)		72.0 (71.0 to 73.0)		0.092†

Abbreviation: CCR8, CC chemokine receptor 8; RFS, recurrence-free survival; ccRCC, clear-cell renal cell carcinoma; ECOG-PS, Eastern cooperative Oncology Group performance status; LVI, lymphovascular invasion; IQR, interquartile range; S, significant (Log-rank test *P* < 0.05); N, non-significant (Log-rank test *P* ≥ 0.05).

Outcome estimation is limited to the largest survival time when it is censored.

* Fisher's exact test to assess the correlation between variables and CCR8.

† Wilcoxon rank-sum test.

‡ Log-rank test of equality of survival distributions for the different levels of CCR8.

### Clinical outcomes and association of CCR8 expression with survival

Median follow-up for patients alive at last follow-up was 73 months (IQR 72-74, range 39-74, *n* = 410). 71 patients (15.0%) recurred during the follow-up including 54 patients (11.4%) who died of RCC. Overall 6-year OS was 86.1% (95%CI, 82.9-89.3) and RFS was 83.0% (95%CI, 79.0-87.0).

Six-year RFS estimates of CCR8^+^ and CCR8^−^ patients were 69.9 (95%CI, 61.3-78.5) and 87.5 (95%CI, 83.4-91.6), respectively (Table 1). In univariate analysis, CCR8 positive expression was significantly associated with worse RFS (*P* < 0.001; Figure 1B), this difference in survival remained significant when restricting analyses to grade 1-2, negative LVI or negative sarcomatoid patients. Furthermore, positive CCR8 expression was an independent predictor of RFS (HR, 2.014; 95%CI, 1.224-3.315; *P* = 0.006) in multivariate analysis. After a 1000-resampled bootstrap correction, it remained its significance (HR, 2.198; 95%CI, 1.154-4.154; *P* = 0.008), together with tumor size, pT stage, Fuhrman grade, LVI and coagulative necrosis (Table 2).

**Table 2:** Proportional hazard model for RFS prediction of non-metastatic ccRCC patients

	Base model		Bootstrap validate model*	
Variables	HR (95% CI)	*P*	HR (95% CI)	*P*
Age at surgery (Continuous by 5-yr increment)	1.090 (0.973 to 1.222)	0.137	Adjusted	
Gender (Male *vs* Female)	1.559 (0.876 to 2.777)	0.131	Adjusted	
ECOG-PS (≥1 *vs* 0)	1.310 (0.671 to 2.556)	0.429	1.444 (0.592 to 3.456)	0.426
Tumor size (Continuous, cm)	1.358 (1.220 to 1.511)	<0.001	1.375 (1.183 to 1.613)	0.003
Pathological T stage (pT1 *vs* pT2 *vs* pT3)		<0.001		0.001
pT2 *vs* pT1	2.034 (0.815 to 5.076)	0.128	1.724 (0.496 to 5.501)	0.226
pT3 *vs* pT1	4.022 (2.042 to 7.920)	<0.001	3.906 (1.428 to 7.996)	0.003
Fuhrman grade (1+2 *vs* 3 *vs* 4)		<0.001		0.001
3 *vs* 1+2	2.206 (1.152 to 4.224)	0.017	2.300 (1.058 to 4.821)	0.018
4 *vs* 1+2	4.038 (1.994 to 8.180)	<0.001	3.985 (1.602 to 9.422)	0.001
LVI (Present *vs* Absent)	2.943 (1.742 to 4.969)	<0.001	2.727 (1.366 to 5.124)	0.002
Sarcomatoid features (Present *vs* Absent)	5.442 (2.060 to 14.374)	0.001	6.250 (1.000 to 37.115)	0.042
Coagulative necrosis (Present *vs* Absent)	2.724 (1.566 to 4.739)	<0.001	2.582 (1.361 to 4.993)	0.002
CCR8 (Positive *vs* Negative)	2.014 (1.224 to 3.315)	0.006	2.198 (1.154 to 4.154)	0.008

Abbreviation: CCR8, CC chemokine receptor 8; RFS, recurrence-free survival; ccRCC, clear-cell renal cell carcinoma; ECOG-PS, Eastern cooperative Oncology Group performance status; LVI, lymphovascular invasion.

* The bootstrap validate model is calculated on the basis of adjusted survival function for age and gender by the time of surgery. Bootstrapping with 1000 resamples were used.

### Predictive impact of CCR8 upon Leibovich score model

**Figure 2:**
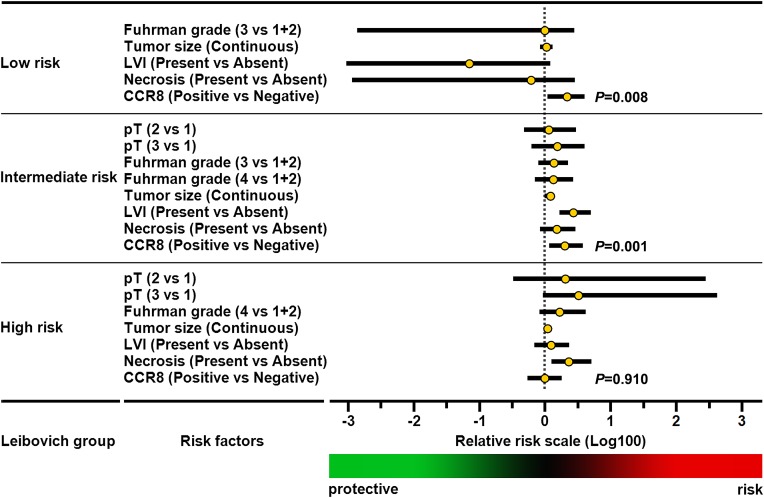
Multivariate analyses of conventional prognostic features in diverse Leibovich risk groups The relative hazard of each feature for recurrence are scaled in logarithmic form, *P*-values of CCR8 expression pertinence to recurrence-free survival are highlighted. Abbreviation: LVI, lymphovascular invasion; RFS, recurrence-free survival; CCR8, CC chemokine receptor 8.

The Leibovich recurrence risk scores of all 472 patients were calculated and divided into three risk groups: low risk (score 0-2; *n* = 260, 55.1%), intermediate risk (score 3-5; *n* = 164, 34.7%), high risk (score≥6; *n* = 48, 10.2%). Kaplan-Meier survival analyses revealed that the diverse outcome between CCR8^+^ and CCR8^−^ patients was dominantly lay in Leibovich low and intermediate risk groups (Log-rank *P* = 0.001, 0.007, respectively; Figure 1C-1E). Stratified multivariate analyses also showed an independent predictive impact of CCR8 for RFS in Leibovich low (HR = 4.616; *P* = 0.009) and intermediate risk groups (HR = 4.002; *P* = 0.006). These data manifested the significant RFS prognoses power of CCR8 in low recurrence risk population of localized ccRCC patients and that this indication was differ from those risk factors composing Leibovich model, including pT stage, Fuhrman grade, tumor size, and necrosis (Figure 2).

### Construction and validation of prognostic nomogram for RFS

**Figure 3:**
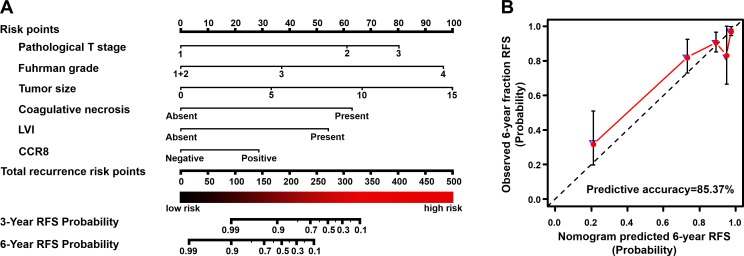
Built-up prognostic nomogram and calibration plots for RFS prediction of postoperative ccRCC patients **A**. Six independent prognostic factors including pathological T stage, Fuhrman grade, tumor size, coagulative necrosis, LVI presentation and CCR8 expression were identified and entered into the nomogram to predict 6-year recurrence risk and recurrence-free survival. **B**. Calibration curves for predicting 6-year RFS of ccRCC patients. The nomogram-predicted RFS is plotted on the x-axis, actual RFS (solid red circles) and bootstrapped RFS (blue hollow triangle) are plotted on the y-axis, a plot along the 45-degree (dash line) indicate a perfect calibration in which the predicted probabilities are identical to the actual outcomes. Abbreviation: LVI, lymphovascular invasion; RFS, recurrence-free survival; CCR8, CC chemokine receptor 8.

A nomogram that incorporated the significant prognostic factors concluded from validated multivariate analyses was established (Table 2 and Figure 3A). Considering the wide variation on confidence interval of sarcomatoid feature, which may resulted from their few effective events, we excluded sarcomatoid feature in the final nomogram without compromising the robustness of the model. The nomogram illustrated pathological T stage, Fuhrman grade and tumor size as sharing the largest contribution to prognostication (weighted ratio = 0.189, 0.237, 0.227, respectively). Intratumoral CCR8 expression showed a moderate impact on outcome (weight ratio = 0.071). Each level of these variables was assigned a score on the scale, and the total score could be easily used to determine the estimated probability of survival at different time point.

The calibration plots presented an excellent agreement in the cohort and a condign consistency in bootstrap resampling analysis between the predicted and actual observation for 3-yr and 6-yr RFS (3-yr RFS data not shown; Figure 3B). In the primary cohort, the Harrell's C-index for the established nomogram to predict RFS (0.854; 95%CI, 0.811-0.896) was significant higher than that of the Leibovich model (0.836; 95%CI, 0.790-0.882; *P* = 0.010), and the AIC was also lower than Leibovich model (706.0 *vs* 738.2). This superior performance of nomogram was also kept significant among TNM I+II patients, UISS model defined and SSIGN score defined low-intermediate risk patients (*P* = 0.044, 0.005, 0.002, respectively; Table 3).

**Table 3:** Prognostication comparison of built-up nomogram and original Leibovich model

		C-index (95%CI)				AIC	
Patients group	No. of patients (%)	Nomogram	Leibovich	Coefficient (95%CI)	*P*	Nomogram	Leibovich
Overall	472 (100%)	0.854 (0.811 to 0.896)	0.836 (0.790 to 0.882)	0.037 (0.013 to 0.061)	0.010	706.0	738.2
TNM stage I+II	345 (73.1%)	0.819 (0.750 to 0.888)	0.768 (0.696 to 0.839)	0.051 (0.001 to 0.101)	0.044	404.8	410.0
UISS low or intermediate risk	438 (92.8%)	0.847 (0.790 to 0.903)	0.797 (0.738 to 0.856)	0.048 (0.015 to 0.082)	0.005	559.7	593.9
SSIGN low or intermediate risk	458 (97.0%)	0.848 (0.796 to 0.900)	0.801 (0.746 to 0.856)	0.049 (0.018 to 0.079)	0.002	608.5	645.0

Abbreviation: C-index, concordance index; AIC, Akaike's information criterion; UISS, UCLA Integrated Staging System; SSIGN, Mayo clinic stage, size, grade, and necrosis; 95% CI, 95% confidence interval.

C-index, 95%CI and AIC are calculated from 1000 bootstrap samples to protect from overfitting.

## DISCUSSION

To date > 5600 separate reports related to RCC outcome prediction have been published. Unfortunately, the prognostic assessment remains controversial and no remarkable progress was made on validating and applying new parameters in routine clinical practice [7]. Many recent prospective studies screened and reported molecular signatures to join existing prognostic models, which represent underlying dysregulation of biological functions associated with disease aggressiveness, including immune response [8, 11, 12]. A good way to select these prognostic factors representing cancer-related immune response may be considering the biological functions of chemokines or cytokines and their sources or targeting cell types, which could help us to subgroup these factors functionally and then select appropriate agents according to their performance.

Chemokine (C-C motif) receptor (CCR8) is one of the novel candidates of tumor prediction because there have been various literatures reported the special behaviors of CCR8 in different pathogenesis. CCR8 was reported to be expressed on Treg cells which may trigger immune inhibition in several inflammatory diseases [14]. Furthermore, evidence rose that endothelial-derived spindle cells of human Kaposi sarcoma acquire chemotaxis through its intrinsic CCR8 expression [15]. The only report that directly indicated the pro-tumorigenic expression of CCR8 on tumor cells was conducted by Das et al, which showed that blocking CCR8 function could inhibits CCR8+ melanoma cell egress from afferent lymphatic into lymph node [5]. However, the expression of CCR8 in RCC remained unclear, despite a recent work showed that up-regulated expression of CCR8 is detected within RCC tissues and primarily limited to CD11b^+^ tumor associated macrophages, while their work enrolled limited 22 RCC patients [10].

In this study, we detected the expression in tumor cells from a segment of non-metastatic ccRCC cases detected and confirmed the intratumoral CCR8 expression in 26% (123 of 472) ccRCC tissue samples, comparative with that of 16% reported by 2013 TCGA cohort data. Analogously, we demonstrated a significant correlation between positive CCR8 expression and the likelihood of post-operative recurrence by competing risk analyses. In order to further strengthen the prognostication impact of CCR8 for clinical recurrence, we developed a post-operative nomogram to predict long-term recurrence-free survival of non-metastatic patients based on 6-year follow-up. The built-up model offer further benefit in addition to the current combined clinicopathologic stratification tools - Leibovich score, which is constructed by pathologic T stage, N stage, tumor size, nuclear grade and histologic necrosis [17]. Of particular interest, we found that this improvement mainly occurred in organ-confined patients or low risk patients defined by UISS and SSIGN models. This may help us to inform postoperative management of early-stage patients for whom the conventional T stage and Fuhrman grades may have weaken prediction effects, as well as to help recognize the patients with low-risk tumor who would ultimately progress and succumb to their disease as an aid of conventional abdomen imaging techniques [18].

As far as we have found, CCR8 is the only C-C motif family chemokine receptor that is dominantly expressed on renal tumor cells instead of infiltrating immune cells while exerting profound impact on recurrence-free survival in localised patients, which means we can easily judge the IHC staining results by positive/negative dichotomy either artificially or digitally, with few effect from intratumoral heterogeneity and immune cell infiltrating susceptibility. This findings may indicated CCR8 as a potential agent candidate of C-C motif chemokine receptor family. It should be acknowledged that this study lack independent external validation, and it seem likely that the study design of retrospectively analyzing post-nephrectomy tumor samples, particularly with the intratumoral heterogeneity of regimens, would hinder the robustness of predictive biomarkers, further experimental studies are also required to identify the detailed role of CCR8 in ccRCC. Nevertheless, we should notice that attempts to identify pharmacological antagonists of CCR8 have been going on, and our work may implicate therapeutic targets of those small molecule inhibitors used on adjuvant therapy for non-metastatic ccRCC [19].

## MATERIALS AND METHODS

### Patient selection

The study database included 472 non-metastatic clear-cell RCC patients from Zhongshan Hospital, Fudan University, Shanghai, China. The primary inclusion criterion were (1) histopathologically-proven clear-cell RCC; (2) received partial- or radical- nephrectomy between Jan 7, 2008 and Dec 23, 2009; (3) had available corresponding archived Formalin Fixed Paraffin Embedded (FFPE) specimen of tumor mass (≥1cm^3^). All these 490 valid patients received surgery after diagnoses, without pre-surgery radiotherapy or chemotherapy. Seven T4 stage patients, seven M1 metastatic patients, and four failed-to-stain cases were then excluded from the cohort, leaving 472 final cases. To ensure consistent data collection, baseline demographic, clinical, and laboratory data were collected simultaneously, MRI and CT scans were reassessed in radiology units, and all archived diagnostic H&E slides were pathologically central reviewed by pathologist (Chen L.) independently. This study was approved by the institutional ethical review boards of hospital and all patients stated informed consent along with phone-call follow-up.

### Data collection

The primary outcome of interest was recurrence-free survival (RFS). RFS was defined as the time from surgery to first renal cell carcinoma recurrence (local or distant metastases, identified by imaging, biopsy, or physical examination). According to clinicians and archived files, occurrences were censored if patients died without clear evidence of recurrence or if the patient was alive at end of follow-up. Follow-up data for all patients were obtained from most recent medical review and ended in Mar 2015. All patients were examined routinely every 5-6 months during the first 5 years of follow-up and annually thereafter. The study did not include any patients with recurrence within 2 months of surgery.

All relevant information on sociodemographic data (age at surgery, gender), pathologic data (pathologic tumor, node, and metastasis status), treatment-related data (type of nephrectomy or chemotherapy). Histological subtypes were re-stratified according to 2014 EAU guidelines [6]. TNM staging was re-classified according to 2010 AJCC TNM classification [20]. Fuhrman grade, LVI, coagulative necrosis, sarcomatoid features were reported according to 2012 ISUP consensus [7]. Eastern ECOG-PS were prospectively recorded and re-archived as previously described [21]. UISS, SSIGN and Leibovich scores were applied to all valid patients according to original scoring algorithm, respectively [17, 22, 23].

### Statistical analysis

Associations between CCR8 expression subgroups *versus* clinicopathologic parameters were evaluated using Fisher exact test and Wilcoxon rank-sum test. RFS was assessed and graphically illustrated using Kaplan-Meier and log-rank test was used for comparing different scoring categories. Independent associations between RFS and assessed clinicopathologic predictors were evaluated using multivariate Cox proportional hazards regression models. Prognostic parameters that were found to be associated with recurrence risk in the development study were further narrowed down to form a nomogram by the strength of the associations with recurrence in both univariate and multivariate analyses. The concordance index (C index) and Akaike's Information Criteria (AIC) were used to assess the predictive accuracy and sufficiency of different models, while Hanley-McNeil test was used to compare between C index [24, 25]. To reduce overfit bias and internally validate the predictive accuracy estimates, multivariable models and C index calculations were subjected to 1000 bootstrap resamples.

Statistical analyses were performed with SPSS, version 21.0 (IBM, Armonk, NY), Stata SE, version 12.1 (Stata, College Station, TX) and R software packages, version 3.1.2 (The R Foundation for Statistical Computing, http://www.r-project.org/). A two-sided *p* value of less than 0.05 was considered to be statistically significant for all reports.

### Procedures

Primary FFPE RCC samples were obtained from the Department of Urology, Zhongshan Hospital with patients' consent and approval of the institutional review board of Fudan University. Microarray development and immunohistochemistry were performed according to the methods previously applied [26] with appropriate antibodies after control staining (anti-CCR8 antibody, ab140796, Abcam, diluted 1/100) (Supplementary Document S1). Immunohistochemistry sections and corresponding H&E sections were scanned by a fully automated microscopy system (Leica DM6000 B, Leica Microsystems GmbH, Mannheim, Germany), images were captured by Leica CV-M2CL camera and analyzed by Leica Ariol 4.0 software automatically. Cases were considered positive for expression when > 10% of tumor cells showed diffuse immunoreactivity.

## SUPPLEMENTARY MATERIAL


