# Supplementary material for: Positive intratumoral chemokine (C-C motif) receptor 8 expression predicts high recurrence risk of post-operation clear-cell renal cell carcinoma patients
Source: Oncotarget. 2015 Dec 24;7(7):8413–21. doi: 10.18632/oncotarget.6761 (PMC4885002; doi:10.18632/oncotarget.6761)
Supplement: Supplementary file 1 [file oncotarget-07-8413-s001.pdf]

Positive intratumoral chemokine (C-C motif) receptor 8 expression predicts high recurrence risk of post-operation clear-cell renal cell carcinoma patients

Supplementary Material

Immunohistochemistry

The FFPE sections were deparaffinized and rehydrated successively though xylene and gradient ethanol (99.9%, 97%, 85%, 70%) incubation at 60℃, then blocked endogenous peroxidase in 3% H2O2 for 30 mins, treated using heat mediated antigen retrieval with sodium citrate buffer (pH6) for 4 mins at 100℃. The section was then block in 10% goat serum for 2h at room temperature, incubated with anti-CCR8, IgG or anti-CCL1 primary antibodies (all diluted 1/100) overnight at 4℃, then incubated with HRP-conjugated secondary antibody for 30 mins at room temperature, and detected using DAB as the chromogen for 2 minutes at room temperature. The sections were finally counterstained with haematoxylin, dehydrate and mounted with DPX. We used TBST (TBS+0.025%Tween-20) to rinse the sections with gentle agitation (3 x10 mins) between these incubation steps.
